# Influence of simulator physical fidelity and immersion on decision-making skill, presence, and cognitive load

**DOI:** 10.1007/s00426-026-02236-2

**Published:** 2026-04-10

**Authors:** Zachariah G. Hoyne, Khaya Morris-Binelli, Sean Müller, Benjamin Piggott, Paola Chivers, Evan Dekker

**Affiliations:** 1 School of Health Sciences, The University of Notre Dame Australia, Fremantle, WA Australia; 2 Centre for Smart Analytics, Federation University Australia, Ballarat, VIC Australia; 3 School of Education, The University of Notre Dame Australia, Fremantle, WA Australia; 4 Institute for Health Research, The University of Notre Dame Australia, Fremantle, WA Australia; 5 School of Medical and Health Sciences, Edith Cowan University, Joondalup, WA Australia; 6 Academic Services and Support Directorate, Federation University Australia, Ballarat, VIC Australia

**Keywords:** Virtual reality, Immersion, Presence, Psychological fidelity, Decision-making

## Abstract

In a variety of domains such as law enforcement and sport, virtual reality is being explored to simulate field-based decision-making contexts. 360-degree video virtual reality (360VR) is innovative as it can immerse the user in a simulated environment, present egocentric, stereoscopic vision and allows for head movements to perceive visual information. However, it is unknown whether physical, rather than psychological fidelity, is crucial for performance, and its impact upon cognitive load, relative to immersion. This study investigated simulator fidelity, presence, and cognitive load across immersion (two-dimensional video and 360VR) in an exemplar sport-specific decision-making task. Thirty skilled Australian Rules Football players completed a task under varied physical to psychological fidelity (normal to blurred vision stimuli). There were no significant differences in decision-making performance across immersion (simulators) but some decrement across vision conditions was observed. Sense of presence was significantly higher in 360VR compared to two-dimensional video, whilst there were no significant differences in cognitive load across simulator modality. These findings suggest that psychological fidelity is crucial for decision-making performance, which is independent of simulator immersion, and ratings of presence and cognitive load. In application, 360VR may be more effective, than video simulation, in increasing user acceptance of the technology to assess and accelerate decision-making skill. Collectively, these findings provide theoretical implications for the presentation of task-relevant visual information over creating simulators which prioritise visual realism.

**Supplementary Information:**

The online version contains supplementary material available at 10.1007/s00426-026-02236-2.

In high-pressure and time-constrained domains, such as sport, military, or law enforcement, perceptual-cognitive-motor skills, which consist of the utilisation of environmental sensory information and prior knowledge to inform action, are crucial for expert performance (Harris et al., 2023; Müller et al., 2023). Two related perceptual-cognitive-motor skills that are vital for superior performance are visual anticipation and decision-making (Williams & Jackson, 2019). Visual anticipation is the pick-up of contextual (e.g., positioning of players on the field of play) and kinematic information (i.e., teammate and opponent movement patterns) to predict an outcome and inform an effective motor response (Morris-Binelli & Müller, 2017; Panchuk et al., 2018). Information picked-up during the anticipatory phase guides decision-making, which is defined as an action choice, such as to pass or maintain possession of the ball in Australian Rules Football (ARF; Piggott et al., 2019). However, assessing this skill in representative field-based settings is logistically challenging, due to physical workload management to minimise injury and busy competition and training schedules (Mangalam et al., 2023; Müller et al., 2015; Nassis et al., 2019). Accordingly, simulators are increasingly being used to investigate decision-making skill (Breed et al., 2018; Robertson et al., 2023).

## Two-dimensional video simulation to assess decision-making

Two-dimensional (2D) video is a common simulation tool to assess decision-making. In sport, videos of simulated field scenarios or match footage are presented to performers through a projector or on a computer, where they make decisions on how best to progress the ball down the field to score (Musculus, 2018; Natsuhara et al., 2020; North & Williams, 2019). Expert decision-making involves recognising and recalling structured defensive and offensive player positioning and predicting players future location on the field (e.g., Berry et al., 2008; Gorman et al., 2011; Williams et al., 2006). Despite the important knowledge that 2D simulation has provided regarding expert decision-making, there are potential technological limitations which may limit its utility to further understand this skill.

Given the dynamic 360-degree nature of many sports, particularly invasion sports such as ARF, 2D simulation may have technological shortcomings that restrict pick-up of perceptual information for superior decision-making skill. First, presenting 2D video on a projector or computer screen limits the performer’s ability to move their head and scan the environment as they would in the real-world, which could influence the capability to pick-up relevant information for decision-making (Craig, 2014; Kittel et al., 2019). Second, many tests are filmed from an elevated and/or third-person point of view with a monoscopic video camera (e.g., Roca et al., 2020; Ward & Williams, 2003) which does not present relevant depth information for motion perception (Howard, 2012; Mütterlein & Hess, 2017), possibly influencing how a performer utilises visual information for decision-making. Therefore, virtual reality (VR) has been proposed to address these potential limitations (Bideau et al., 2010; Craig, 2014; Düking et al., 2018; Le Noury et al., 2022).

## Advantages of virtual reality simulation

VR is an immersive technology which presents information to users via a head-mounted display (HMD; Müller et al., 2023). Two common types of VR include animated virtual reality (AVR) which presents a synthetic and interactive environment to the user (Le Noury et al., 2021). Alternatively, 360-degree virtual reality (360VR) involves use of a 360-degree video camera to capture and present real-world footage. Unlike AVR, 360VR does not allow the user to interact with and manipulate the simulated environment. However, one significant benefit of 360VR is that it is more cost-effective, as specialist expertise is not required to create the simulated environment (Müller et al., 2023). Accordingly, 360VR is increasingly being used to investigate decision-making skill in sport (see Faure et al., 2020 for a review).

The capability of 360VR to (i) present first person (ego-centric) stereoscopic vision, (ii) allow the user to move their head to view the simulation environment, and (iii) present the simulated environment in a HMD that occludes the real world, is proposed to increase the user’s level of immersion and presence (Düking et al., 2018). Immersion is defined as the physical masking of the real world (Mütterlein & Hess, 2017) and is thought to benefit decision-making by limiting distractions and helping to create a sense of presence in the simulated environment (Harris et al., 2020; Ochs & Sonderegger, 2022). Presence is more subjective and refers to feelings of being transported to and engaging with the simulated environment (Barfield et al., 1995; Mütterlein, 2018). An increased sense of presence is believed to benefit decision-making performance as the feeling of being transported into a representative environment may facilitate decision-making processes that more closely match those implemented during real competition (Harris et al., 2020).

Despite the potential benefits of 360VR over 2D simulation, to our knowledge, there is only one study which has compared decision-making performance between 360VR and 2D simulation in an invasion sport. Kittel et al. (2019) assessed elite and amateur ARF umpires’ decision-making performance and perceived ecological validity (i.e., how closely the presented environment replicated the real world) in a 360VR and 2D video simulator. They found elite umpires’ decision-making significantly outperformed amateur umpires in both simulators. Interestingly, elite and amateur umpires rated the 360VR environment as having significantly higher ecological validity than 2D video. Although these findings suggest that 360VR may better replicate the real-world performance environment, decision-making performance was not directly compared between both simulators. Therefore, the influence of immersion on decision-making skill is largely unknown. Further limitations such as the use of a monoscopic 360-degree video camera, presentation of elevated third person footage in the 2D video task and ego-centric vision in the 360VR task, and no measurement of presence makes it difficult to compare decision-making performance between each modality. Therefore, additional research is needed to further understand whether decision-making performance is different in 360VR compared to 2D video simulators and whether it is influenced by immersion, presence, and the provision of depth information. Beyond these technological factors that can influence perception, the design and implementation of 360VR should consider psychological factors to ensure the simulated environment targets underlying mechanisms of decision-making skill.

## Psychological vs. physical fidelity

Psychological fidelity refers to the degree to which a simulation reproduces the perceptual-cognitive mechanisms of expert skill (Harris et al., 2020; Müller et al., 2023). Physical fidelity, on the other hand, pertains to the realism of the simulated environment, including visual display resolution, field of view, and object behaviour (Harris et al., 2020). Despite increased discussion and evidence that psychological fidelity, rather than physical fidelity, is crucial decision-making, VR design often prioritises physical fidelity (Champion et al., 2023; Müller et al., 2023). For instance, opinion papers suggest that for a VR system to be representative, perception-action coupling needs to be maintained (Janssen et al., 2023). However, empirical evidence indicates that decision-making skill is not dependent upon overt perception-action coupling (Brault et al., 2012; Kalén et al., 2021; Ranganathan & Carlton, 2007; Vignais et al., 2015). The crucial design feature is to ensure representative perceptual information, rather than high physical fidelity of stimuli (Kalén et al., 2021; Müller et al., 2023). Therefore, psychological fidelity should be the focus for assessment of expertise in VR systems.

An example of the importance of psychological fidelity for expert decision-making is the application of blur to the visual field during sport decision-making tasks. This reduces superficial visual information (e.g., logos on players shirts) whilst preserving key relational kinematic movement patterns between teammates and opponents. Despite the impoverished visual display, research using 2D video simulation has demonstrated that expert athletes can maintain superior and above chance level decision-making performance compared to their lesser-skilled counterparts (Jackson et al., 2009; Ryu et al., 2015). Further, training under blur in 2D video can enhance decision-making performance to a greater extent than training under normal vision (van Biemen et al., 2018). These studies indicate that highly skilled individuals rely on relational information (pattern perception) based upon less superficial visual information to make superior decisions. In VR, however, only one study has utilised blur to investigate expert performance in sport (Limballe et al., 2022). Limballe et al. (2022) presented expert combat athletes with an AVR boxing environment that had light, medium, and strong levels of blur. Interception performance did not deteriorate following medium blur manipulations, which is consistent with studies utilising blur in 2D video. As this study used an AVR environment, there is currently no evidence of the influence of blur on decision-making performance in 360VR and how this compares to 2D video. Further, as presence was not measured in this study, it is unknown whether implementing blur reduces presence (as physical fidelity is reduced), and whether this in turn influences decision-making performance. Another important psychological factor to consider when investigating decision-making in 360VR is cognitive load.

## Cognitive load and simulator design

As 360VR is suggested to have an increased capability to create representative simulated environments compared to 2D video, it is possible that this could influence cognitive load, and therefore, decision-making performance (Champion et al., 2023; Harris et al., 2020). Cognitive load theory proposes that working memory capacity is limited and features of the task will influence the amount of this capacity utilised in performance (Paas et al., 2003; Sweller et al., 1998). Cognitive load comprises of intrinsic (task load), extraneous (method of task presentation), and germane (cognitive resources for schema utilisation) components, which together reflect the demands placed on working memory (Paas et al., 2003; Sweller, 2010). High intrinsic or extraneous load can impair performance by limiting capacity for germane load to utilise schemata (Paas et al., 2003). Interestingly, the immersive nature of HMDs used to increase presence is proposed to increase cognitive load (Ochs & Sonderegger, 2022). However, there has been scant research exploring how increases in immersion and presence of VR simulators influence cognitive load and decision-making skill. Moreover, the influence of immersion and presence on cognitive load and decision-making skill across simulator physical/psychological fidelity (normal display to blur) is unknown. Such an investigation could provide important insights into how to utilise immersive technology, such as 360VR, to assess, and potentially enhance, decision-making skill, whilst considering the broader cognitive workload of athletes (Champion et al., 2023).

## The present study

The purpose of this study was to investigate the influence of immersion (360VR and 2D video) and psychological fidelity (normal and blurred vision) on presence, decision-making performance, and cognitive load in the exemplar invasion sport of ARF. Participants were required to watch representative ARF play sequences and decide which teammate they would pass the ball. They viewed play sequences under normal or blurred vision conditions either in a HMD or on a projected video display. Based upon the previously discussed literature, the following hypotheses were formulated. First, it was predicted that presence would be rated higher in 360VR compared to 2D video. Second, because presence, immersion, and stereoscopic depth perception are critical features of VR, it was predicted that decision-making performance would be higher in 360VR compared to 2D video. Third, it was predicted that decision-making performance will progressively decrease from no blur to higher blur conditions, but not to a degree that decision-making is at chance level. Fourth, it was predicted that presence would be rated higher in the no blur condition compared to the blurred conditions, with presence rated higher in 360VR for all vision conditions compared to 2D video. Finally, it was predicted that cognitive load would be rated higher in 360VR compared to 2D video and will progressively increase with greater visual blur.

## Method

### Participants

Thirty skilled male ARF players (*M*_age_ = 23.13, *SD* = 4.07, *M*_years played_ = 15.17, *SD* = 5.18) were recruited for this study. Participants either played ARF in a semi-professional state (provincial) or local amateur league. Based upon the framework by McKay et al. (2022), these participants can be classified as highly trained (Tier 3, semi-professional state level competition; *n* = 15) and trained (Tier 2, local-level representation; *n* = 15) players. All participants had to meet the following criteria to participate: (i) not be colourblind, (ii) have normal or corrected-to-normal vision, and (iii) not suffer from vertigo. If participants had recently suffered a concussion, they were not able to participate in the study until 12 days had passed from concussion diagnosis as per concussion protocols (McCrory et al., 2017; Pearce et al., 2015), and they received medical clearance to partake in sport.

An a-priori power analysis was conducted in G*Power (Version 3.1.9.7) for the comparison of decision-making performance in 360VR and 2D video. This analysis indicated that for a repeated measures analysis with α = 0.05, 80% power, and 95% confidence interval, 30 participants with 96 trials per task modality (192 total trials per participant) could detect a small effect of *f* = 0.08 (Morris-Binelli et al., 2021). In addition, a-priori power analyses for the comparison of presence and cognitive load across 360VR and 2D video tests indicated that 30 participants could detect a small effect size of *f* = 0.18, with α = 0.05, 80% power, and 95% confidence interval. Finally, a-priori power analysis for the chance comparisons indicated for 30 participants, a moderate effect size of *d* = 0.53 could be detected, with α = 0.05, 80% power, and 95% confidence interval. Ethical approval for this study was granted by the lead authors institutional ethics committee (Reference Number: 2022–140F). Participants provided written informed consent prior to participating.

### Creation of the decision-making task

**Fig. 1:**
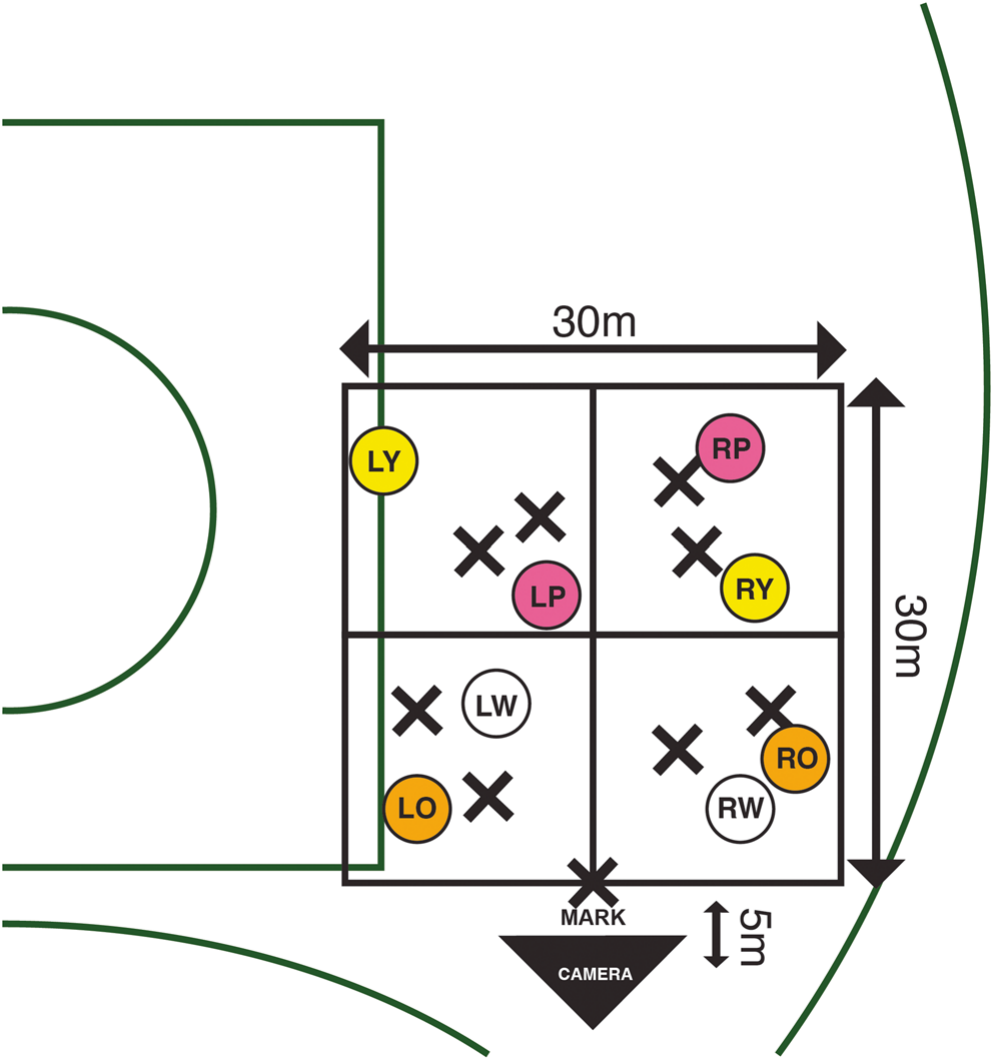
Schematic of the decision-making task on the field of play. X marks denote the defending players and circles denote attacking players. LW = left white, LO = left orange, RW = right white, RO = right orange, LP = left pink, RP = right pink, LY = left yellow, RY = right yellow

In collaboration with three expert ARF coaches (Level 3 Accredited), the research team devised an ARF decision-making task which targeted mark-disposal decision-making. These scenarios included eight attacking players and eight defending players performing different running patterns in a 30 × 30 m zone that was located on the side of the centre square (located in the middle of the ARF playing field) between the two 50 m arches of an ARF field. The playing area was further divided into four sub-zones, with each containing two attacking and two defending players (see Fig. 1). The defending players (including the player standing the mark) wore dark clothes to ensure a clear visual distinction between the attacking players who wore light coloured clothing and either a white, orange, pink, or yellow singlet. The three expert coaches scored each scenario from the best decision (8 points) to the worst decision that the player in possession of the ball could make (1 point) to progress the ball down the field to score (see Supplementary Material A for further details of the decision-making task development). As per other research that has investigated decision-making in sport (e.g., Murr et al., 2021; Piggott et al., 2019), the three coaches discussed the scoring of each scenario until 100% agreement was reached.

To obtain the video footage to create the decision-making task, a 360-degree stereoscopic video camera (Vuze 3D 360 4K VR Camera, Humaneyes Technologies Ltd., Neve Ilan Israel) sampling at 30 frames-per-second was fastened atop of a tripod at a height of 1.7 m and 5 m from the intersection of the front playing zones, perpendicular to the opposition (defending) player standing the mark (see Fig. 1). This filming position simulated the viewing perspective of an ARF player (i.e., the decision-maker) who had marked the ball. During filming, the attacking players were instructed to run and call for the ball as if the 360-degree camera was their teammate in possession of the ball, whilst the defending players defended an attacking player. To capture adequate video footage to create the decision-making task, eight unique versions of the eight scenarios were filmed. This resulted in a pool of 64 unique video clips from which to create the test and familiarisation trials based upon those that the coaches and research team deemed adequate for the test. The quality of each trial in terms of whether the attacking and defending players correctly executed the running patterns with match-like intensity was assessed by the expert coaches and research team. For this experiment, 31 unique video clips which met this quality assessment were used to create the test matrix, familiarisation trials, and practice trials, with the same trials presented to all participants (see Supplementary Material B for details of how the 360-degree videos were processed).

#### 360VR and 2D video test creation

**Fig. 2:**
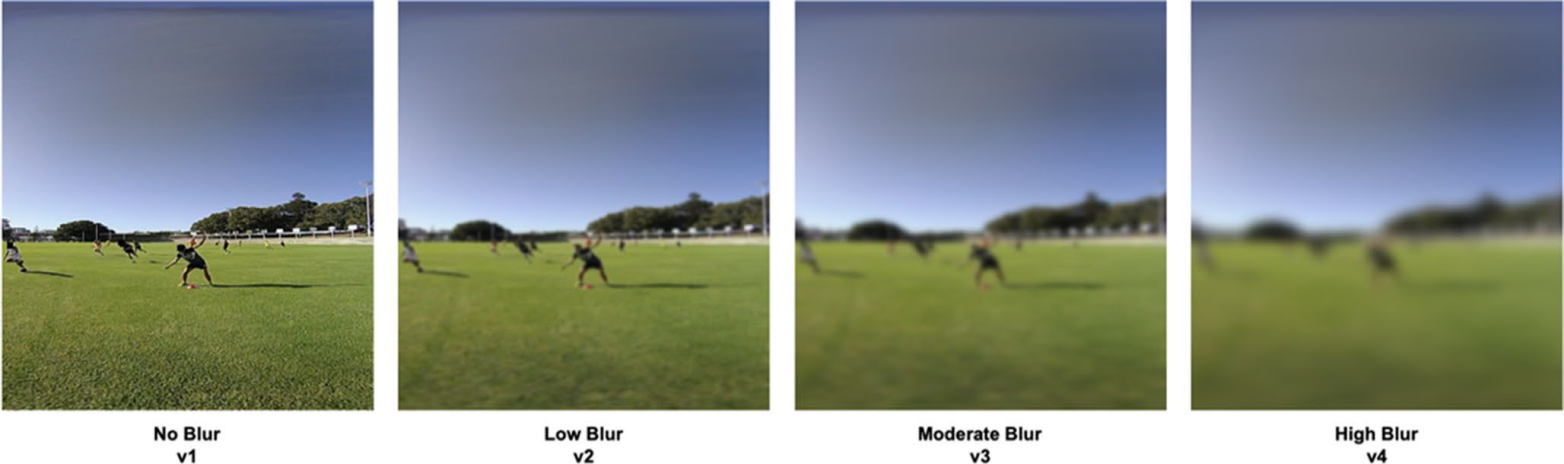
Change in visual clarity across vision conditions

The test matrix for the 360VR and 2D video task each consisted of 8 scenarios × 3 unique versions × 4 vision conditions = 96 trials. The same unique versions of each scenario were presented in the 360VR and 2D video tasks. Familiarisation trials consisted of 8 scenarios × 4 vision conditions. In addition, four practice trials were created which presented each vision condition to participants. The video footage used to create the familiarisation and practice trials was different to the footage included in the test proper. All audio captured during filming was removed from the test, familiarisation, and practice trials, as the focus was upon visual information. To target a key mechanism of expert decision-making, and to address one of the main research questions, a blur filter was applied to blocks of test trials. The following visions conditions were created: (i) a normal vision condition with no blur filter (v1), (ii) a low blur condition that had 10 units of VR blur (v2), (iii) a moderate blur condition that had 20 units of VR blur (v3), and (iv) a high blur condition that had 40 units of VR blur (see Fig. 2). Supplementary Material C provides details of how the blur levels were created and calibrated against previous research.

In the 360VR task, trial numbers were displayed for 1 s in front and to the left and right of the viewing perspective of the participant with the front number in yellow for participant orientation. Conversely, the 2D video test only displayed the trial number in yellow in the centre of the screen. Thereafter, a 1 s still image of the first frame of the scenario was displayed for participant orientation, followed by the entire scenario clip (~ 6–8 s). A 5 s intertrial interval (ITI) occurred between each trial which contained an audio cue at four seconds to alert participants of the approach of the next trial. To minimise order effects, each vision condition block of 24 trials was counterbalanced using a Latin Squares approach. Further, to reduce familiarisation with the test stimuli, the presentation of scenarios was randomised across the 360VR and 2D video tasks as well as within each vision condition. Therefore, four counterbalanced decision-making tasks were created which were presented in 360VR and 2D video.

### Procedure

Participants individually completed the 360VR and 2D video tasks in a counterbalanced order. In 360VR, participants wore a Meta Quest 2 (Meta, California, United States of America) HMD to view the decision-making task. The HMD was wirelessly displayed onto a laptop computer (MacBook Pro 18.3, Apple Inc., California, United States of America) and subsequently projected onto a screen. This allowed the researcher to view the participant’s perspective and record their verbal responses via a video camera (GoPro HERO9, GoPro Inc., California, United States of America) for later coding. Verbal responses are deemed to be an acceptable discriminator of expert decision-making performance (Aglioti et al., 2008; Kalén et al., 2021). At the beginning of each testing session, participants completed the pre-installed “First Steps” (Oculus, Meta, California, United States of America) tutorial game for 5 minutes to help orient them to VR. Thereafter, participants viewed the familiarisation and practice trials before beginning the test proper. For 2D video, the task was played in VLC Media Player (Version 3.0.18, VideoLAN Association). This program was used as it can display 360VR video files in a 2D format. The 2D video task was presented to the participants via a projector that was connected to a laptop computer onto a screen 150” (3.81 m diagonal) in size. Participants stood a mathematically calculated distance of 2 m away from the wall to replicate the viewing angle (19°) in a match. At the beginning of the 2D video task, a keyframe image was presented in order to zoom out the display so that participants could view the entire playing area without distorting the scene.

Participants were informed that the aim of the decision-making task was to choose the best decision and that the speed of their response did not affect the score awarded. Accordingly, participants were free to respond at any stage during the trial, but were reminded to respond before the next trial began. On each trial, participants verbalised their decision of which teammate to pass the ball to (i.e., “left orange”, “left white”, “right orange”, “right white”, “left pink”, “left yellow”, “right pink”, or “right yellow”), even if they were completely uncertain. If participants did not provide a response on a given trial, they were marked as making the worst decision (i.e., score of 1). Participants completed a sense of presence and cognitive load questionnaires after each vision condition. No feedback was provided to participants during the test proper.

The total time taken to complete both the 360VR and 2D video tasks, including the questionnaires, was approximately 90 min (~ 45 min for each modality). Participants completed the 360VR and 2D video tasks in two separate sessions, which were separated by ~ 24 days. This was due to difficulties accessing participants in a single session of ~ 90 min before their usual ARF training due to work and/or study commitments. Not interrupting participants’ usual ARF training was important to obtain access to skilled ARF players.

### Dependent measures and statistical analyses

#### Sense of presence

To measure the dependent variable of sense of presence, participants completed the Independent Television Commission-Sense of Presence Inventory (ITC-SOPI; Lessiter et al., 2001) after each vision condition in both modalities. The inventory uses a five-point Likert scale ranging from (1) strongly disagree to (5) strongly agree to measure four factors: (a) spatial presence (feeling of being within the simulated environment), (b) engagement (being involved with, and enjoyment of, the simulated environment), (c) ecological validity (lifelikeness and realness of the simulated environment), and (d) negative effects (feelings of dizziness, nauseousness and eyestrain within the simulated environment). For spatial presence, engagement, and ecological validity, higher scores (i.e., closer to 5 on the 5-point Likert scale) signify greater levels of these factors. For negative effects, lower scores (i.e., closer to 1 on the 5-point Likert scale) indicate fewer negative effects. The ITC-SOPI is a reliable method of measuring presence in immersive environments with high Cronbach’s alpha scores across all factors (spatial presence = 0.94, engagement = 0.89, ecological validity = 0.76, and negative effects = 0.77; Lessiter et al., 2001).

Four separate Generalised Estimating Equation (GEE) analyses were run in SPSS (IBM SPSS statistics, Version 27, IBM Corp., New York, United States) to examine differences in spatial presence, engagement, ecological validity, and negative effects across the independent variables. Modality (360VR, 2D video) and vision condition (v1, v2, v3, v4) were included as fixed effects and individual participants were included as a repeated factor. As spatial presence, engagement, ecological validity, and negative effects are continuous measures, linear GEEs were performed and residuals were checked to be within ± 3.29 for skewness and kurtosis (Ballinger, 2004; Field, 2018). GEEs were used as they facilitate the correct modelling of repeated observations and allow for non-normal distribution models (Ghisletta & Spini, 2004). For these analyses, any significant main and interaction effects were followed up using Bonferroni corrected pairwise comparisons and the alpha level was set at 0.05.

#### Decision-making performance

The dependant variable of decision-making performance was analysed on its original 1–8 scale. Therefore, to compare decision-making performance across modality and vision condition, a mixed effects Tobit regression (metobit) was performed in STATA Statistical Software (Version 18, StataCorp, 2023). Modality and vision condition were included as main effects and as a two-way interaction, whilst individual participants were included as a repeated factor. A Tobit regression was performed rather than an ordinal GEE as it allows for the identification of relationships between censored dependent variables to a set of independent variables (Williams, 2016). Accordingly, the metobit was bound by the possible score for each trial of 1 to 8. Significant main and interaction effects were investigated using pairwise comparisons with a Bonferroni correction and the alpha level was set at 0.05. For ease of interpretation, all descriptive statistics (e.g., means, standard errors) are reported as percentage scores relative to the maximum possible score (i.e., 8 out of 8 = 100%). To determine whether decision-making performance was significantly above, at, or below the 12.5% guessing level for an eight-choice task, Wilcoxon one sample signed-rank tests were performed. These tests were used as normality checks indicated the majority violated skewness and kurtosis of ± 1.96 (Field, 2018). For these tests, alpha level was adjusted to 0.01 to guard against familywise error (Field, 2018). Cohen’s *r* effect sizes were calculated for Wilcoxon one sample signed-rank tests to indicate the magnitude of decision-making response accuracy above chance, with *r* =.10, *r* =.30, and *r* =.50 indicating a small, medium, and large effect, respectively (Cohen, 2013).

#### Cognitive load

To measure the dependant variable of cognitive load, participants completed the Rating Scale for Mental Effort (RSME; Zijlstra, 1993) after each vision condition for each modality. The RSME is a one-dimensional linear scale that ranges from 0 to 150, with 0 indicating no effort at all, 75 indicating moderately effortful, and 150 indicating very effortful. This scale has been validated and is a reliable measure of cognitive load that has been used in many domains, such as sport, aeronautics, and healthcare (Alimohammadi et al., 2019; Ghanbary Sartang et al., 2016; Runswick et al., 2018; Veltman & Gaillard, 1996).

Differences in cognitive load were also examined using a GEE. Modality and vision condition were included as a fixed effect and individual participants as a repeated factor. Again, linear GEEs were performed, and residuals were checked to be within ± 3.29 for skewness and kurtosis (Ballinger, 2004; Field, 2018). Any significant main and interaction effects were followed up using Bonferroni corrected pairwise comparisons (α = 0.05).

## Results

### Sense of presence

The GEE analysis indicated no significant two-way interaction between modality and vision condition on any of the subscales of the ITC-SOPI [spatial presence, ꭓ^2^ (3) = 0.49, *p* =.921; engagement, ꭓ^2^ (3) = 0.93, *p* =.818; ecological validity, ꭓ^2^ (3) = 6.89, *p* =.075; negative effects, ꭓ^2^ (3) = 5.29, *p* =.152].

**Fig. 3:**
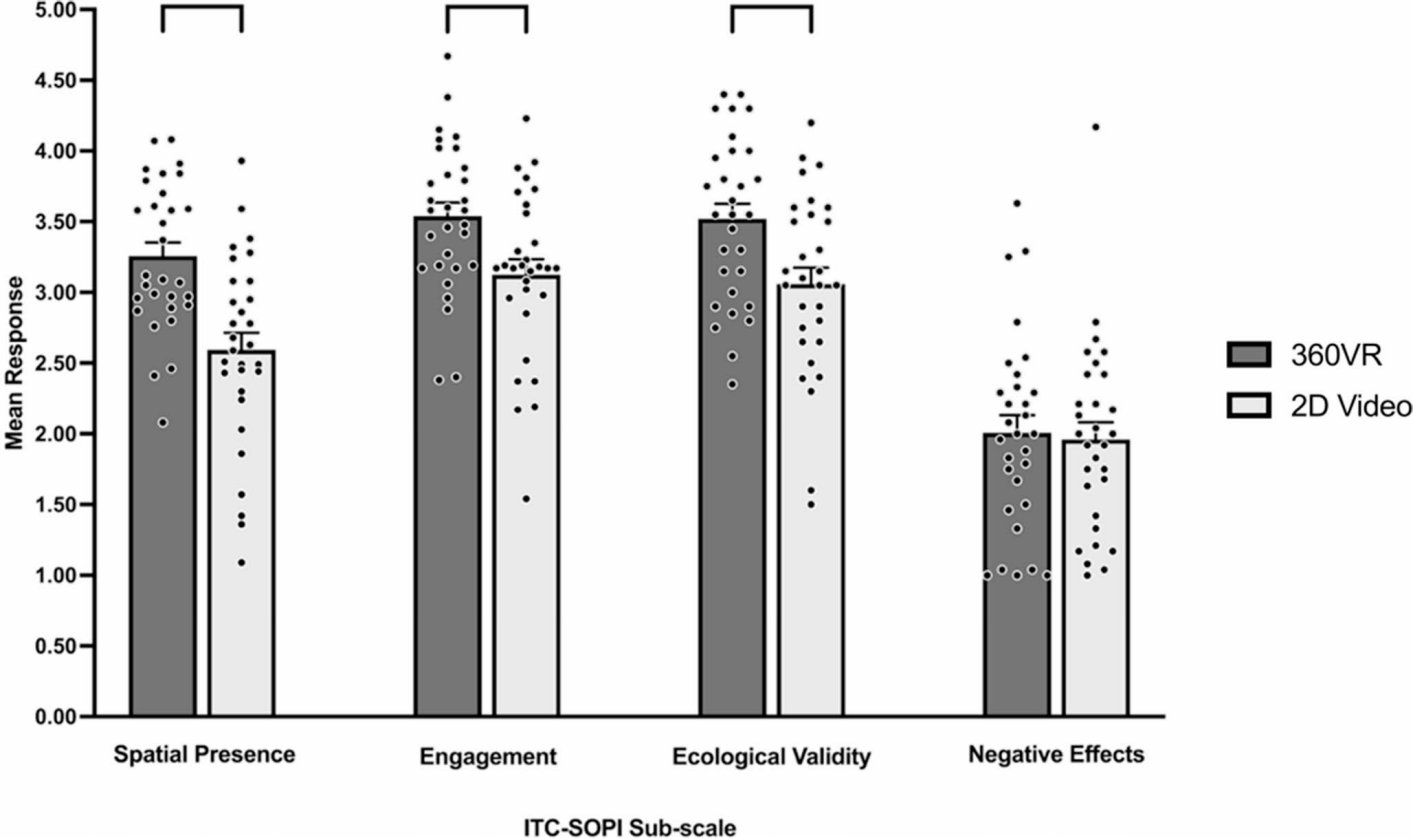
Sense of presence across task modality. Bracketed groupings indicate significant difference between modalities (*p* <.05). Error bars indicate standard error. Individual participant data points are represented by dots

GEE analyses indicated that the main effect of modality significantly influenced sub-scales of the ITC-SOPI, with spatial presence [360VR, *M* = 3.26, *SE* = 0.09, 2D video, *M* = 2.59, *SE* = 0.12, ꭓ^2^ (1) = 32.19, *p* <.001], engagement [360VR, *M* = 3.54, *SE* = 0.09, 2D video, *M* = 3.13, *SE* = 0.11, ꭓ^2^ (1) = 19.33, *p* <.001], and ecological validity [360VR, *M* = 3.52, *SE* = 0.11, 2D video, *M* = 3.06, *SE* = 0.12, ꭓ^2^ (1) = 15.62, *p* <.001] significantly higher in 360VR compared to 2D video (see Fig. 3). In contrast, there was no significant difference in negative effects between 360VR (*M* = 2.01, *SE* = 0.12) and 2D video (*M* = 1.96, *SE* = 0.12), ꭓ^2^ (1) = 0.21, *p* =.646 (see Fig. 3).

**Fig. 4:**
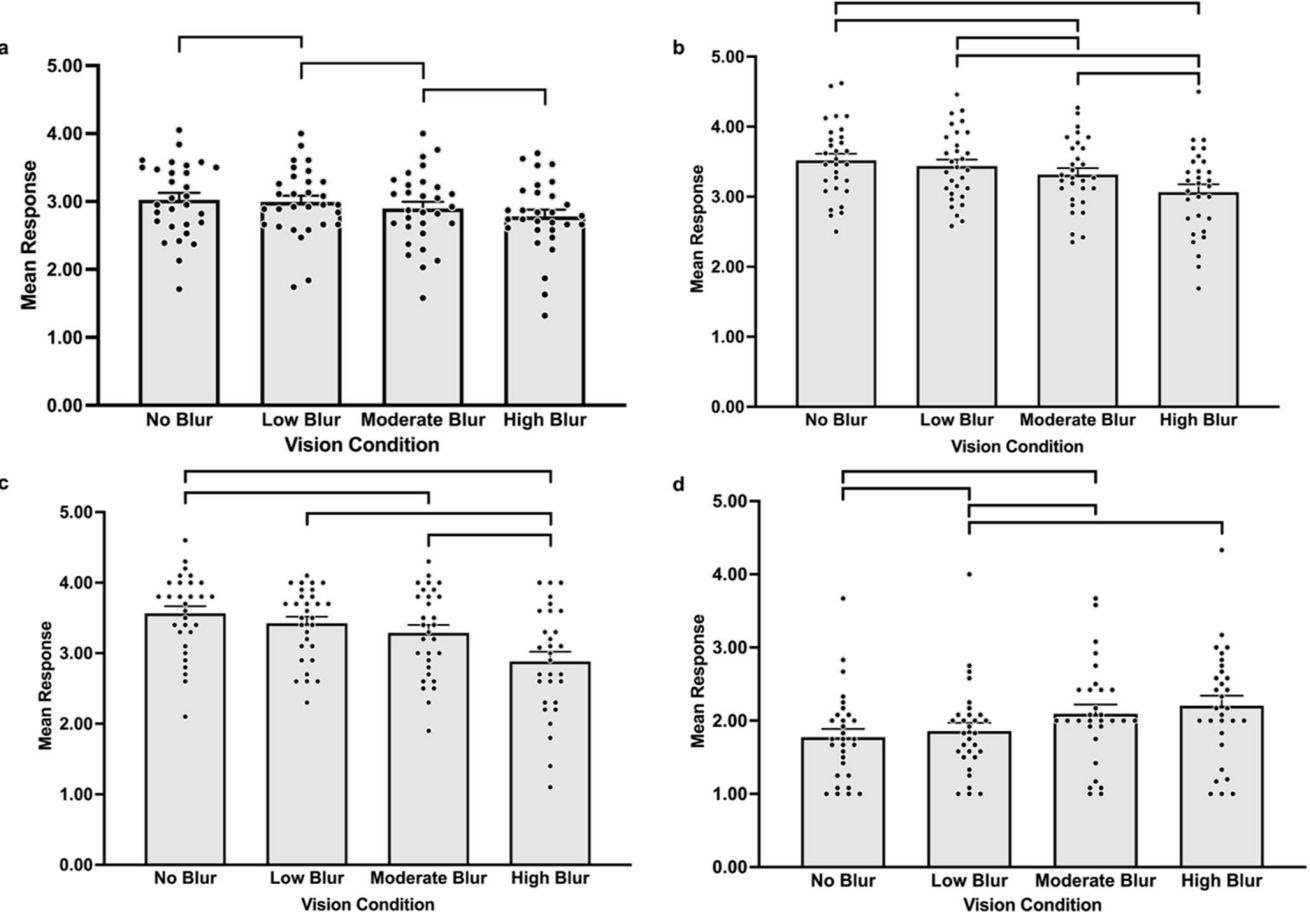
Spatial presence (**a**), engagement (**b**), ecological validity (**c**), and negative effects (**d**) across vision conditions. Bracketed groupings indicate significant pairwise comparisons across vision conditions (*p* <.05). Error bars indicate standard error. Individual participant data points are represented by dots

GEE analyses found the main effect of vision condition significantly influenced sense of presence, with significant differences across vision conditions observed for spatial presence [no blur, *M* = 3.03, *SE* = 0.10, low blur, *M* = 2.99, *SE* = 0.09, moderate blur, *M* = 2.90, *SE* = 0.10, high blur, *M* = 2.78, *SE* = 0.10, ꭓ^2^ (3) = 18.86, *p* <.001], engagement [no blur, *M* = 3.52, *SE* = 0.10, low blur, *M* = 3.44, *SE* = 0.09, moderate blur, *M* = 3.32, *SE* = 0.09, high blur, *M* = 3.06, *SE* = 0.11, ꭓ^2^ (3) = 29.99, *p* <.001], ecological validity [no blur, *M* = 3.56, *SE* = 0.10, low blur, *M* = 3.42, *SE* = 0.09, moderate blur, *M* = 3.29, *SE* = 0.11, high blur, *M* = 2.88, *SE* = 0.14, ꭓ^2^ (3) = 28.08, *p* <.001], and negative effects [no blur, *M* = 1.77, *SE* = 0.11, low blur, *M* = 1.86, *SE* = 0.11, moderate blur, *M* = 2.09, *SE* = 0.12, high blur, *M* = 2.20, *SE* = 0.14, ꭓ^2^ (3) = 31.68, *p* <.001]. Specifically, spatial presence was rated significantly lower in high blur compared to moderate blur (*p* =.026), low blur (*p* <.001), and no blur (*p* =.001). Engagement was rated significantly higher in no blur compared to moderate (*p* = 002) and high blur (*p* <.001), as well as low blur being rated significantly higher than moderate blur (*p* =.026) and high blur (*p* <.001). Finally, moderate blur was rated significantly higher than high blur (*p* <.001). Ecological validity was rated significantly higher in no blur compared to moderate blur (*p* =.011) and high blur (*p* <.001) and was rated significantly higher in low blur and moderate blur compared to high blur (*ps* < 0.001). Compared to no blur, negative effects were rated significantly higher in moderate blur and high blur (*ps* < 0.001). Furthermore, negative effects, when compared to low blur, were rated significantly higher in moderate blur (*p* =.013) and high blur (*p* =.001). Figure 4 presents mean responses to the sub-scales of the ITC-SOPI across vision conditions, as well as significant pairwise comparisons.

### Decision-making performance

Tobit regression indicated no significant two-way interaction between modality and vision condition on decision-making performance, ꭓ^2^ (3) = 2.81, *p* =.422.

**Fig. 5:**
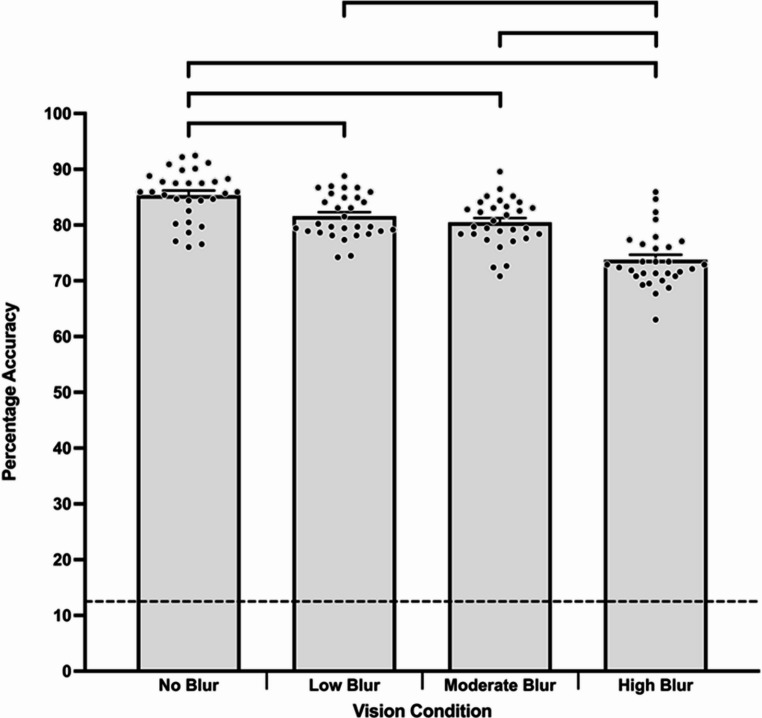
Mean decision-making response accuracy across vision conditions. Bracketed groupings indicate significant differences between vision conditions (*p* <.05). Dashed line denotes the guessing level (12.5%). Error bars indicate standard error. Individual participant data points are represented by dots

Tobit regression did reveal a significant main effect of vision condition on response accuracy, ꭓ^2^ (3) = 81.88, *p* <.001. In comparison to no blur (*M* = 85.33%, *SE* = 0.58%), response accuracy was significantly lower in low blur (*M* = 81.63%, *SE* = 0.64%, *p* <.001), moderate blur (*M* = 80.50%, *SE* = 0.65%, *p* <.001), and high blur (*M* = 73.75%, *SE* = 0.71%, *p* <.001). Response accuracy was also significantly higher in low blur compared to high blur (*p* <.001). Further, response accuracy was significantly greater in moderate compared to high blur (*p* <.001; see Fig. 5). Wilcoxon one sample signed-rank tests indicated that response accuracy was significantly above the guessing level of 12.5% in the no blur (*r* =.87), low blur (*r* =.87), moderate blur (*r* =.87), and high blur (*r* =.87) vision conditions (*p*s < 0.001).

### Cognitive load

GEE revealed no significant two-way interaction between modality and vision condition on cognitive load, ꭓ^2^ (3) = 2.67, *p* =.445. GEE also revealed modality did not significantly influence cognitive load (360VR, *M* = 75.85, *SE* = 0.55; 2D video, *M* = 75.84, *SE* = 0.52), ꭓ^2^ (1) = 0.00, *p* =.997.

**Fig. 6:**
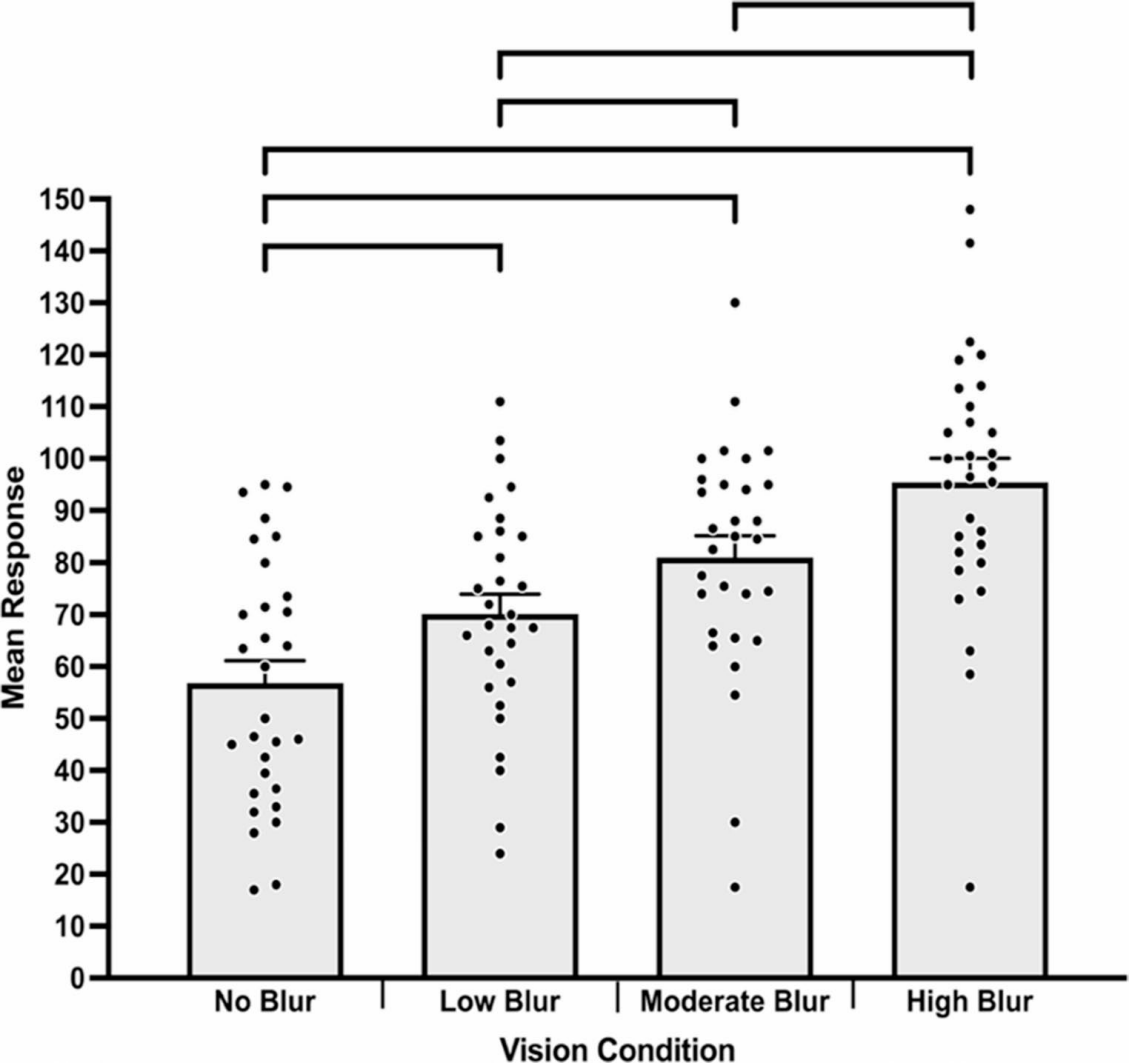
Cognitive load across vision conditions. Bracketed groupings indicate significant differences between vision conditions (*p* <.05). Error bars represent standard error. Individual participant data points are represented by dots

GEE for vision condition indicated this variable significantly impacted cognitive load, ꭓ^2^ (3) = 78.75, *p* <.001. The source of the main effect was that cognitive load was significantly lower in the no blur condition (*M* = 56.82, *SE* = 0.66) compared to the low (*M* = 70.13, *SE* = 0.59), moderate (*M* = 81.02, *SE* = 0.63), and high (*M* = 95.42, *SE* = 0.72) blur conditions (*p*s < 0.05). Cognitive load was also significantly lower in low compared to moderate and high (*p*s = 0.001) blur conditions. Moreover, cognitive load was significantly lower in moderate compared to high blur conditions (*p* <.001; see Fig. 6).

## Discussion

The purpose of this study was to investigate the influence of immersion, as well as physical and psychological fidelity, on presence, decision-making, and cognitive load in the exemplar invasion sport of ARF. Accordingly, skilled ARF players completed a domain-specific decision-making task under normal and varied degrees of blurred visual stimuli in both 360VR and 2D video. The findings of this study provide important theoretical and practical information for the design and implementation of 360VR to assess, and potentially enhance, decision-making skill in sport, and other domains, where accurate decisions are crucial for performance.

### Sense of presence

It was predicted that immersion would influence perceptions of presence. Results across spatial presence, engagement, and ecological validity sub-scales of the ITC-SOPI supported this hypothesis as all were rated significantly higher in 360VR compared to 2D video. This finding aligns with the proposition that more immersive environments result in higher perceptions of being *within* the simulated environment (spatial presence), greater user engagement, and a simulated environment perceived to better represent the real word (Harris et al., 2020; Mütterlein, 2018). These findings build upon previous studies in sport which have compared presence across different task modalities. Loiseau Taupin et al. (2023) found novice boxers who had to move their heads to dodge incoming punches from a virtual opponent whilst wearing a HMD, rated the 360-video task as having a significantly higher presence than the 2D video task. However, as both the tasks in Loiseau Taupin et al. (2023) were presented in a HMD, the current study’s findings provide unique knowledge that perceived presence is significantly higher in 360VR when presented in a HMD compared to a 2D video presented via a projector; the latter of which is more commonly used to investigate decision-making in sport. Further, the finding that ecological validity is perceived by performers as higher in 360VR compared to 2D video aligns with Kittel et al. (2019), who reported ARF umpires rated a 360VR decision-making task as having higher ecological validity than the same task presented in 2D video from an elevated broadcast position. Crucially, however, as our study’s task presented the same viewing position across modalities, our findings provide new knowledge that ecological validity is enhanced by viewing 360-degree footage in a HMD. A possible reason for this is unlike in 2D video, our 360VR task presented stereoscopic vision to participants, which is proposed to be important to create simulated environments that represent the real world (Craig, 2014; Faure et al., 2020). However, as our study did not compare presence across two immersive tasks, which either did or did not present stereoscopic vision, this conclusion should be met with caution and could be an interesting avenue for further research. In addition, we found no significant differences in negative effects between 360VR and 2D video. This is encouraging for the uptake and acceptance of 360VR for users who may be apprehensive about spending time in an HMD due to the possibility of cybersickness (Faure et al., 2020).

Another aim of this study was to investigate the influence of blur, and consequently, the reduction of physical fidelity (normal to blur conditions), on presence across 360VR and 2D video. Overall, results support the hypothesis that perceptions of presence will be affected by blur as participants’ ratings of spatial presence, engagement, and ecological validity progressively decreased, whilst ratings of negative effects progressively increased, as blur levels increased. In contrast to our predictions, however, immersion did not influence sense of presence relative to the vision conditions. As this is the first study to our knowledge to investigate presence across varied degrees of blurred visual displays between 360VR and 2D video simulators, it is difficult to compare our findings to previous work. Nonetheless, the current findings provide unique knowledge that across 360VR and 2D video simulators, and therefore higher and lower immersion, respectively, the clarity of the visual display influences performers’ perceived level of presence within the environment (spatial presence), as well as their engagement with the task, and how ecologically valid that task is perceived. Interestingly, there were no significant differences on any of the sub-scales of the ITC-SOPI between the no blur and low blur conditions. This could be because even though the physical fidelity of the environment was reduced in the low blur condition, there was still a sufficient level of psychological fidelity present to maintain perceptions of presence. Accordingly, as psychological fidelity relates to the degree to which the simulation replicates the perceptual-cognitive demands of the real-world task (Harris et al., 2020; Müller et al., 2023), it is possible that the low blur condition replicated the demands experienced in a match, hence the lack of a decrease in presence.

### Decision-making performance

An important finding in the current study was the lack of significant differences in decision-making across the 360VR and 2D video tasks. This finding did not support our hypothesis and contradicts one of the main arguments as to why VR is a more suitable technology than 2D video to investigate perceptual-cognitive-motor skills. That is, the greater immersion and presence created for the performer in VR creates a simulated environment that better corresponds to the real-world task (Drew, 2021; Faure et al., 2020; Harris et al., 2020). However, despite the 360VR task resulting in significantly greater spatial presence, engagement, and ecological validity than the 2D video task, the lack of performance differences indicates that these factors, whilst likely important for increasing users’ positive perceptions of the simulator, have little influence on decision-making skill. Comparison of our findings to other invasion sport studies is challenging due to the dearth of literature in this area. The only other study did not directly compare decision-making performance between 360VR and 2D video and used different test stimuli and viewing perspectives across both tasks (Kittel et al., 2019). Nonetheless, the descriptive statistics for the elite umpires in Kittel et al. (2019) suggest little difference in decision-making between 360VR and 2D video, which aligns with our findings. Interestingly, our findings contrast those of Discombe et al. (2022) who, in the striking sport of cricket, reported batters anticipated deliveries more accurately when viewing the test stimulus via a 360-degree video in a HMD compared to 2D video via a projector. A potential reason for this contrasting finding could be due to different perceptual-cognitive demands between the invasion sport of ARF and the striking sport of cricket, which may elucidate differences in simulator modality on performance. Further, Discombe et al. (2022) included only amateur participants, which could indicate that simulator type may not influence perceptual-cognitive-motor skill once a certain level of expertise is reached.

In relation to blur, findings indicated that across 360VR and 2D video, response accuracy in the no blur condition was significantly better than in the low, moderate, and high blur condition (see Fig. 5). Despite this decrease in decision-making accuracy, performance was significantly above the guessing level across all vision conditions, and the magnitude of performance change from no blur to high blur could be considered relatively low (11.57%, see Fig. 5). These findings support our hypothesis and somewhat align with Ryu et al. (2015), who reported skilled participants response accuracy in a 2D video decision-making task significantly decreased when moderate and high blur was introduced (Ryu et al., 2015). Further, despite this drop in response accuracy, decision-making performance across their blur conditions was significantly above guessing level. Direct comparison of our findings to the only study to investigate the influence of blur on perceptual-cognitive-motor skill in VR is challenging as Limballe et al. (2022) implemented gaze-contingent blur in a AVR boxing task. Nonetheless, their findings indicated that expert boxers could maintain interception performance when central or peripheral vision is blurred. Accordingly, our findings build upon Limballe et al. (2022) to demonstrate that in 360VR (and 2D video), blurring of the whole visual field decreased to an extent decision-making response accuracy, but the manipulation did not appear to significantly impede the capability of skilled performers to pick-up information for decision-making. Collectively, our findings along with others (Jackson et al., 2009; Limballe et al., 2022; Ryu et al., 2015), reinforce the importance of psychological fidelity (i.e., the presentation of domain-specific perceptual information) rather than physical fidelity (e.g., visual clarity) for expert perceptual-cognitive-motor skill (Harris et al., 2020; Müller et al., 2023).

### Cognitive load

In contrast to our predictions, the results of this study revealed no significant difference in cognitive load between 360VR and 2D video. This is an interesting finding as the increase in immersion and presence in 360VR compared to 2D video is proposed to result in an increase in extraneous cognitive load (Harris et al., 2020). There is evidence that inexperienced participants who undertook surgery skills training report higher cognitive load when completing this training in head mounted immersive VR compared to 2D video (Frederiksen et al., 2020). As ARF players in the current study had considerable experience in ARF, our findings provide tentative evidence that increased immersion may not result in greater cognitive load for skilled performers.

The current findings also indicated that cognitive load significantly increased as the visual display became more severely impoverished (see Fig. 6), which aligns with our hypothesis. Greater cognitive load as blur increased appeared to contribute, at least in part, to a decrease in response accuracy (see Fig. 5). This potential explanation aligns with previous research which has reported increases in cognitive load result in decreased anticipation accuracy in experienced soccer players (Alder et al., 2021; Gredin et al., 2020). The current findings also provide new insights into the influence of impoverished visual displays on cognitive load. As cognitive load significantly increased across vision conditions (see Fig. 6), participants likely increased concentration and attentional focus to pick-up task relevant perceptual information in an attempt to maintain decision-making performance. Interestingly, although the progressive increase of blur did not substantially decrease decision-making performance to guessing level, it did almost double the reported value of mental effort. Therefore, reducing the physical fidelity of a simulator whilst maintaining psychological fidelity, appears to significantly influence cognitive load. This is likely an important factor to consider when designing tasks to assess, and potentially enhance, perceptual-cognitive-motor skills (Champion et al., 2023).

### Theoretical, technological, and practical implications

There are two main theoretical implications from our study. First, subjective ratings of presence that decreased relative to immersion did not affect performance across simulator modality. This indicates that presence and performance are two separate psychological constructs that act independently of each other irrespective of simulator immersion. Accordingly, fundamental principles that underpin virtual reality simulator design need to consider that increasing immersion through a virtual reality simulator will increase perceived transportation to an alternative environment (Mütterlein, 2018). However, such transportation to an immersive environment did not correspond to increased performance in our study. A possible reason for this discrepancy is that presence in a virtual environment is likely prominent immediately when the HMD is first worn by the user (Ochs & Sonderegger, 2022). During the trial the user has to selectively attend to irrelevant/relevant visual-perceptual information to inform decision-making, with evidence from a memory learning task that immersion can cause distraction, compared to two-dimensional video displays (Ochs & Sonderegger, 2022). Second, high physical fidelity in terms of standard photorealistic displays do not seem to be the only visual information necessary to maintain performance above guessing level. This indicates that psychological fidelity of simulating opponent relational information in biological movement patterns is the most crucial for decision-making performance (Jackson et al., 2009; Ryu et al., 2015). This is consistent with the initial work of Johansson (1973) and colleagues (Runeson & Frykholm, 1983) who reported that biological motion can be perceived through point-light displays that present only kinematic, and not, superficial, information. Indeed, representative task-specific perceptual information is argued to be a crucial aspect of perceptual-cognitive-motor skill, such as decision-making, across different theories of motor learning and control, such as cognitive theories (Schmidt, 2003), ecological dynamics (Gibson, 1979), and predictive processing (Friston, 2005; Rao & Ballard, 1999). The findings of this study reiterate the importance of this visual-perception information for skilled performance and highlight the need for three-dimensional virtual reality and 2D video simulation displays to target psychological fidelity, rather than an overemphasis on visual physical fidelity (Müller et al., 2023).

These theoretical implications have an important technological implication. Where financial resources may limit purchase of a 360VR camera to create an immersive display, an alternative to use a lower cost standard video camera will not impede open-field-play decision-making performance, and likely, its acceleration. That stereoscopic vision simulated through 360VR did not result in superior performance to 2D video indicates that depth perception was not a crucial factor at least for open-field-play player positional change-based decision-making in this study. It appears that relational kinematic information is preserved across stereoscopic (360VR) to monoscopic (2D) simulators resulting in similar performance. This is likely because depth information can also be gleaned from two-dimensional sources (Miles et al., 2012). Therefore, 2D video simulation is still valuable to assess and accelerate decision-making performance, but 360VR can be used to boost user engagement with the task.

In relation to practical implications, we recommend that sports teams and other organisations carefully consider why they may want to invest in virtual reality and its underpinning psychological fidelity. Our research group has previously commented on the importance of virtual reality simulator design to include perceptual information required for expert anticipation and decision-making (Müller et al., 2023). For example, this includes point-light displays of opponents, which can now, in our laboratory, be modelled with custom designed software to create single and multiple opponents to be representative of one-on-one striking and open field-play sports, respectively. As long as psychological fidelity has been considered from the perspective of perceptual information that guides anticipation and decision-making, it is then possible for the user to make responses in a perception-only or perception-action coupled manner based upon the capabilities of the technology (Müller et al., 2023). Ultimately, by targeting this key mechanism underpinning performance, there is a greater likelihood that user’s perceptual-cognitive-motor skill can be assessed, and then trained, in an evidence-based manner. Failure to carefully design virtual reality simulators can be significantly detrimental to performance (Müller et al., 2023).

## Future research, limitations, and conclusions

A limitation of this is study is that it focused solely upon the use of visual information for decision-making. However, a systematic approach was needed to first investigate whether immersion and physical/psychological fidelity influenced presence, decision-making performance, and cognitive load. Having addressed these variables it is then possible to increase representative task design through simulation that incorporates multi-sensory information. A further limitation relates to the physical representativeness of these simulators, as neither provided opportunities for actioned-based responses. Yet, there is evidence that decision-making skill is not dependent on overt perception-action coupling (Ranganathan & Carlton, 2007; Vignais et al., 2015), and verbal responses activate motor regions in the brain, whilst discriminating expert performance (Aglioti et al., 2008; Kalén et al., 2021).

Future research could systematically simulate visual, contextual, and auditory information to investigate how they progressively inform and differentiate expertise across 360VR and 2D simulators. Knowing how multi-sensory information informs expertise in decision-making, relative to immersion, and presence, will provide a comprehensive understanding of virtual reality simulation. Indeed, some studies have investigated use of multi-sensory information, such as visual information with contextual (e.g., opponent action tendencies), auditory (e.g., teammate informing a performer where to place the ball) and/or tactile (e.g., bat vibrations when hitting a ball) information in 2D video (e.g., Cañal-Bruland et al., 2018; Gray, 2009; Klatt & Smeeton, 2020) and VR (e.g., Helm et al., 2020). Despite these studies indicating multi-sensory information use is important for informing anticipation and decision-making, no studies to our knowledge have compared use of multi-sensory information across VR and 2D video simulators. After such investigation, progression can be made to examine which sensory modalities need to be modelled in 360VR and 2D video simulators, and together whether available sensory information and simulator type enhances decision-making skill. It could also be worthwhile for future research to investigate whether 360VR has any benefits over 2D video in opposition and performance analysis, where team structures, set plays, or defensive positioning could be reviewed and taught in an immersive environment that enhances engagement. Additional qualitative investigations will also be important to better understand perceived benefits, usability, and the real-world values of virtual reality from the perspective of coaches and athletes.

Virtual reality simulators offer users exciting and engaging experiences. As the technology evolves and becomes more accessible, 360VR may become more than an instrument to transport the user into an alternative environment. However, the findings from this paper cast some doubt as to whether stereoscopic virtual reality simulation provides any benefit over 2D video in the assessment of decision-making. This does not suggest that designing decision-making instruments to be engaging is not valuable. In fact, this might be *the* key advantage of 360VR over traditional 2D video, whereby users want to be in an HMD to assess and accelerate their perceptual-cognitive skills. While the possible benefit of virtual simulation on training perceptual-cognitive skill is in its infancy, if sports coaches or instructors in other domains can create opportunities for users to partake in simulated decision-making training that is engaging, representative, and targets psychological fidelity through virtual reality, it may be invaluable to accelerate performance without the increased risk of injury (Mangalam et al., 2023). Having addressed fundamental questions of 360VR simulator design relative to decision-making performance, we can now move forward to further explore its value to accelerate perceptual-cognitive-motor skill.

## Supplementary Information

Below is the link to the electronic supplementary material.

Supplementary file 1 (DOCX 36.6 KB)

## Data Availability

The data that support the findings of this study are available upon reasonable request from the corresponding author. The data are not publicly available due to their containing information that could compromise the privacy of research participants.

## Declarations

### Public significance statement

This study investigated key concepts related to virtual reality and video simulators on expert decision-making skill. High visual clarity and immersion within a 360VR simulator appeared to have limited influence on decision-making performance. However, engagement in 360VR was higher than in video simulation. Therefore, 360VR might be more engaging for training decision-making.
