# Supplementary material for: Influence of simulator physical fidelity and immersion on decision-making skill, presence, and cognitive load
Source: Psychol Res. 2026 Apr 10;90(2):72. doi: 10.1007/s00426-026-02236-2 (PMC13068698; doi:10.1007/s00426-026-02236-2)
Supplement: Supplementary file 1 — (DOCX 36.6 KB) [file 426_2026_2236_MOESM1_ESM.docx]

# Supplementary Material

**Associated Manuscript**: Influence of Simulator Physical Fidelity and Immersion on Decision-Making Skill, Presence, and Cognitive Load

**Authors**: Hoyne, Z.G., Morris-Binelli, K., Müller, S., Piggott, B., Chivers, P., and Dekker, E.

**Journal**: Psychological Research

## A. Mark-Disposal Decision-Making and Scenario Development

In Australian Rules Football (ARF), kicking the ball to a teammate who then catches the ball on the full (known as a mark), is one of the main attacking strategies employed in the game. During this time, opposition players are unable to tackle the player in possession of the ball or encroach on a protected area, which extends 10 m either side of the player with the ball through to the player on the mark. After the ball is marked, the player in possession of the ball has a limited amount of time (approximately 6-8 seconds; Appleby & Dawson, 2002) to identify a teammate to dispose the ball to, and therefore, move the ball down the field. Once this period has elapsed, the umpire calls “play on” and opposition players are free to tackle the player with the ball to dispossess them of it and gain possession of the ball for their team to try to score (Australian Football League, 2025). The time period of a player marking the ball until the time when play on is called is the decision-making window that our task targeted.

In two sessions, the coaches, who had extensive knowledge of sports-specific play patterns that occur in semi-professional ARF, met with the research team to devise eight different mark-disposal decision-making scenarios. The coaches discussed common play patterns that defending and attacking players perform in this match play scenario on this location of the field until they agreed upon eight scenarios that they believed captured common player movements.

## B. 360-Degree Video Processing

After filming, the video files were transferred from the 360-degree video camera to a laptop computer (MacBook Pro 18,3, Apple Inc., California, United States of America) where they were processed in Vuze VR Studio (Humaneyes Technologies Ltd., Neve Ilan, Israel). The files were rendered as a 360-degree video at 16:9 aspect ratio, standard stitching, no stabilisation, H.264 codec, auto optimal bitrate, and equirectangular over/under with stereo audio. The videos were then transferred to Adobe Premiere Pro (v23.1.0, Adobe Inc., California, United States of America) in order to create the ARF decision-making video tasks.

## C. Blur Calibration

Although previous research has investigated the influence of blur on decision-making in sport using a 2D video paradigm (e.g., Ryu et al., 2015), information surrounding specific and transferable blur amounts to apply to our task were not easily attainable. Therefore, the research team created a computer script that can objectively and universally measure the degree of blur that an image contains across different blur formulae. With this program, comparison of blur using a Laplacian approach was performed on images containing different levels of Gaussian blur filter used by Ryu et al. (2015) to 2D and 360-degree images from our task that were blurred using the pre-installed VR Blur Immersive Video Effect in Adobe Premiere Pro. The computer script extracts the high-frequency components of the image (edges), and then by computing the variance of the Laplacian response (mean), provides a single number which quantifies the blurriness of the image. This comparison guided how much blur was applied in the low, moderate, and high blur conditions so that the vision conditions in our study closely matched those utilised in Ryu et al. (2015).

# References

Appleby, B., & Dawson, B. (2002). Video analysis of selected game activities in Australian rules football. Journal of Science and Medicine in Sport, 5(2), 129-142. https://doi.org/10.1016/s1440-2440(02)80034-2

Australian Football League. (2025). Laws of Australian football. https://www.afl.com.au/about-afl/laws-of-the-game

Ryu, D., Abernethy, B., Mann, D. L., & Poolton, J. M. (2015). The contributions of central and peripheral vision to expertise in basketball: How blur helps to provide a clearer picture. Journal of Experimental Psychology: Human Perception and Performance, 41(1), 167-185. https://doi.org/10.1037/a0038306
